# Effects of neuraxial analgesia technique on labor and maternal–fetal outcomes: a retrospective study

**DOI:** 10.1007/s00404-022-06600-6

**Published:** 2022-05-22

**Authors:** Cecilia Lazzari, Ricciarda Raffaelli, Roberto D’Alessandro, Chiara Simonetto, Mariachiara Bosco, Pier Carlo Zorzato, Stefano Uccella, Fabrizio Taddei, Massimo Franchi, Simone Garzon

**Affiliations:** 1 Department of Obstetrics and Gynecology, AOUI Verona, University of Verona, Verona, Italy; 2 Department of Obstetrics and Gynecology, Santa Chiara Hospital, APSS Trento, Trento, Italy; 3 Department of Anesthesia and Intensive Care, AOUI Verona, University of Verona, Verona, Italy

**Keywords:** Labor analgesia, Labor duration, Combined spinal epidural analgesia, Epidural analgesia, First stage of labor, Maternal–fetal outcomes

## Abstract

**Purpose:**

To compare the effects of epidural analgesia (EA) and combined spinal epidural analgesia (SEA) on labor and maternal–fetal outcomes.

**Methods:**

We retrospectively identified and included 1499 patients with a single cephalic fetus who delivered at the study center from January 2015 to December 2018 and received neuraxial analgesia at the beginning of the active phase of labor (presence of regular painful contractions and cervical dilatation between 4 and 6 cm). Data including analgesia, labor characteristics, and maternal–fetal outcomes were retrieved from the prospectively collected delivery room database and medical records.

**Results:**

SEA was associated with a shorter first stage of labor than EA, with a median difference of 60 min. On multivariable ordinal logistic regression analysis, neuraxial analgesia, gestational age, fetal weight, labor induction, and parity were independently associated with the first stage length: patients in the EA group were 1.32 times more likely to have a longer first stage of labor (95% CI 1.06–1.64, *p* = 0.012) than those in the SEA group. Additionally, a significantly lower incidence of fundal pressure was performed among patients who underwent SEA (OR 0.55, 95% CI 0.34–0.9, *p* = 0.017). No associations were observed between the used neuraxial analgesia technique and other outcomes.

**Conclusions:**

SEA was associated with a shorter length of the first stage of labor and a lower rate of fundal pressure use than EA. Further studies confirming the effects of SEA on labor management and clarifying differences in maternal–fetal outcomes will allow concluding about the superiority of one technique upon the other.

**Supplementary Information:**

The online version contains supplementary material available at 10.1007/s00404-022-06600-6.

## Introduction

Neuraxial analgesia is a safe and effective pain control method recommended by the World Health Organization (WHO) for healthy pregnant women who request pain relief during labor [1–3]. The literature agrees that maternal request is a sufficient indication in the absence of any medical contraindication [4, 5]. Consequently, neuraxial analgesia use increased worldwide in the past three decades, although the percentage of women receiving neuraxial analgesia varies among different countries and depends on several factors, such as cultural beliefs and local medical practice [6].

The most frequently used techniques for neuraxial analgesia are epidural analgesia (EA) and combined spinal epidural analgesia (SEA). In EA, drugs are administered only in the epidural space through a peridural catheter, whereas in SEA, epidural analgesia follows an intrathecal injection of opioids and/or local anesthetics. A 2012 Cochrane systematic review concluded that SEA, compared with low-dose EA, provides slightly faster pain relief with no difference in overall maternal satisfaction, although associated with higher rates of itching. No differences were noted concerning the mode of delivery, need for labor augmentation, maternal complications, and neonatal outcome [7]. Nevertheless, the evidence comparing EA and SEA is limited. Indeed, the widespread use of pain control during labor with neuraxial analgesia introduced a new model of obstetric care, providing changes in the manner of assisting labor and in the characteristics of delivery itself based on the observation that labor analgesia affects labor and delivery [8, 9]. However, whether EA and SEA have similar or different effects on labor and delivery, such as differences in labor length, is still under-investigated [10]: it has been hypothesized that SEA may be associated with a shorter first stage, due to the faster onset of analgesic effect and more rapid drop in epinephrine [11]. Based on this scenario, the present study aimed to compare the effects of these two techniques (SEA versus EA) on labor characteristics and maternal–fetal outcomes.

## Materials and methods

We retrospectively reviewed the prospectively collected data from the delivery room database at the Department of Obstetrics and Gynecology of the AOUI Verona, University of Verona, Verona, Italy. The database prospectively collects demographic, clinical, pregnancy, delivery, and newborn data regarding all patients who deliver after 23 0/7 weeks of gestation. Moreover, detailed information about the neuraxial analgesia is prospectively collected for each patient by the anesthesiologists dedicated to the delivery room in a separate and cross-matched database.

We retrieved and reviewed the records of all women who delivered between January 2015 and December 2018. For this retrospective study, we identified and included all women with a single cephalic fetus who received neuraxial analgesia at the beginning of the active phase of labor (defined as the presence of regular painful contractions and cervical dilatation between 4 and 6 cm). We excluded those patients who denied research consent for data collection and analysis for research purposes.

Demographic, clinical, pregnancy, delivery, analgesia, and newborn data were retrieved from the database. Specifically, we retrieved data about maternal age, body mass index, ethnic group, parity, gestational age, cervical dilatation at the diagnosis of labor, length of the first stage of labor (from the diagnosis to the complete cervical dilatation of 10 cm), administration of oxytocin during the first stage of labor, length of the transition phase (from the complete cervical dilatation of 10 cm to the presence of maternal urge to push) and of the second stage of labor (from the beginning of maternal urge to push to the delivery of the fetus), mode of delivery (cesarean section [CS], instrumental delivery, vaginal delivery), total blood loss after delivery, development of severe (third and fourth degree) vaginal tear, adoption of fundal pressure maneuver, the use of episiotomy, newborn weight, APGAR score at the 1st and 5th minute of life, and fetal arterial biochemistry. Details regarding neuraxial analgesia were retrieved from the dedicated prospective database using the unique patient number. Any missed information in the databases was obtained by reviewing electronic medical charts records.

At our hospital, the progression of labor is evaluated through obstetric visits carried out every 2 h or sooner if necessary. According to our internal labor protocol, when cervical dilation was slower than 2 cm in 2 h, amniorrhexis and/or oxytocin were considered and administered.

The indication for labor analgesia at the beginning of the active phase of labor (inclusion criteria) is mainly based on maternal request. Neuraxial analgesia is the only medical analgesia available at our center; the choice of EA or SEA is based on the anesthesiologist’s preference and performed per the hospital protocol. In the EA, the epidural block was performed using a 17G Tuohy needle using the loss of resistance technique, with a 19G open tip epidural catheter (Arrow^®^); ropivacaine 0.1% with sufentanil 10 mcg in 15 mL of saline was used. The SEA was performed by needle-through-needle technique (a 17G Tuohy epidural needle and 25G Sprotte spinal needle) for the intrathecal injection followed by placement of the epidural catheter, and ropivacaine 2 mg with sufentanil 2.5 mcg in 5 mL of saline was administered. For both techniques, needles were inserted into the L2–3 or L3–4 interspaces with the patient in a sitting position, and following the initial bolus, analgesia was carried out in the same way for both groups. Analgesia during the first stage of labor was conducted with ropivacaine 0.1% in association with sufentanil 5 mcg in 15 mL of saline until top-ups on request when visual analogue scale (VAS) was > 4. In the second stage of labor, all women received ropivacaine 0.13% plus sufentanil 5 mcg in 15 mL of saline on request (VAS score > 4).

Patient, pregnancy, and fetal characteristics, along with the labor data and obstetrics outcomes, were summarized using standard descriptive statistics and compared between patients who received EA and SEA. Non-normally and normally distributed variables were analyzed using the Mann–Whitney *U* test and *t* test, respectively. Categorical variables were analyzed using the Chi-squared test or Fisher’s exact test as appropriate. We investigated the association between the type of neuraxial analgesia and labor, maternal, and newborn outcomes using univariate and multivariable ordinal, logistic, or linear regression analysis, as appropriate. A two-tailed *p* value < 0.05 was considered statistically significant. Statistical analysis was performed using STATA/SE 16.1 for Mac (Intel 64-bit) Revision 21 Jan 2021.

## Results

From January 1, 2015 through December 31, 2018, 2777 (27.3%) women received neuraxial analgesia during labor out of 10,187 women who delivered at the Department of Obstetrics and Gynecology of the AOUI Verona, University of Verona, Verona, Italy. Among them, 881/2777 (31.7%) received neuraxial analgesia during the latent phase of labor (absence of regular painful contractions or cervical dilation < 4 cm), and 397/2777 (14.3%) patients received the neuraxial analgesia at a more advanced stage of labor (cervical dilation between 7 and 10 cm or second stage). We identified and included 1499/2777 (54%) patients with a single cephalic presenting fetus who received the neuraxial analgesia at the beginning of the active phase of the first stage of labor (presence of regular painful contractions and cervical dilatation between 4 and 6 cm): 402/1499 (26.8%) patients underwent EA and 1097/1499 (73.2%) patients SEA.

Demographic, clinical, and pregnancy characteristics of the study population are summarized in Table 1, overall and stratified based on the type of neuraxial analgesia. No statistically significant differences were noted in maternal age, obesity, gestational age, ethnicity, parity, and obstetric complications in the two groups. A higher proportion of inductions of labor was observed in the EA group (150; 37.3%) as compared to the SEA group (341; 31%), (*p* = 0.032).

**Table 1:** Demographic, clinical, and pregnancy characteristics of the study population, overall and stratified based on the type of neuraxial analgesia

Variable	Total (1499)	EA (402)	SEA (1097)	*p *value
Age (years, SD)	32.3 (5.03) Missing: 0	32.2 (5.1)	32.3 (5)	0.807
BMI ≥ 30 (*n *%)	74 (4.9) Missing: 0	20 (5)	54 (4.9)	0.967
Gestational age (weeks, IQR)	40 (39–40.75) Missing: 0	40 (39–40.75)	40 (39–40.75)	0.281
Post term pregnancies (*n* %)	643 (43.45) Missing: 0	179 (44.5)	464 (42.3)	0.381
Sub-Saharan Africa (*n *%)	Missing: 0	5 (1.2)	24 (2.2)	0.693
Asiatic (*n* %)		7 (1.7)	22 (2)	
Centre-South America (*n *%)		8 (2)	15 (1.4)	
Middle East/North Africa (*n *%)		12 (3)	34 (3.1)	
Caucasian (*n* %)		370 (92)	1002 (91.3)	
Multiparous (*n *%)	318 (21.2) Missing: 0	76 (18.9)	242 (22.1)	0.186
Obstetrical complications (*n* %)	288 (19.2) Missing: 0	76 (18.9)	212 (19.3)	0.855
Hypertensive disorder (*n* %)	82 (5.5) Missing: 0	28 (7)	54 (4.9)	0.123
Diabetes (*n* %)	97(6.5) Missing: 0	22 (5.5)	75 (6.8)	0.342
FGR (*n* %)	22 (1.3) Missing: 0	8 (2)	14 (1.3)	0.309
pPROM (*n* %)	13(0.9) Missing: 0	1 (0.2)	12 (1.1)	0.205
Oligohydramnios (*n* %)	70 (4.7) Missing: 0	22 (5.5)	48 (4.4)	0.372
Polyhydramnios (*n* %)	22(1.5) Missing: 0	3 (0.7)	19 (1.7)	0.225
Cholestasis (*n* %)	8 (0.5) Missing: 0	0 (0)	8 (0.7)	0.118
Fetal malformations (*n* %)	12 (0.8) Missing: 0	2 (0.5)	10 (0.9)	0.532
Induction of labor (*n* %)	491 (32.7) Missing: 0	150 (37.3)	341 (31.1)	0.023
Induction with oxytocin (*n* %)	491(32.7) Missing: 0	11 (7.3)	20 (5.9)	0.498
Induction with prostaglandins (*n* %)		124 (82.7)	298 (87.4)	
Induction with amniorrhexis (*n* %)		11 (7.3)	16 (4.7)	
Induction with amniorrhexis and oxytocin (*n* %)		4 (2.7)	7 (2)	

*SD* standard deviation, *BMI* body mass index, *IQR* interquartile range, *FGR* fetal growth restriction, *pPROM* premature prelabor rupture of membranes, *EA* epidural analgesia, *SEA* spinal epidural analgesia

Data regarding labor characteristics are reported in Table 2. The adoption of SEA was associated with a statistically significant shorter duration of the first stage of labor compared to that observed in the EA group (EA 300, 180–420 min; SEA 240, 150–390 min; *p* = 0.004). The difference between medians of first stage length in the two groups was 60 min. SEA was also associated with a lower use of oxytocin augmentation of the first stage of labor. No statistically significant differences were observed between the two groups regarding transition phase, second stage, or total labor length, as well as no statistically significant differences were noted in the use of amniorrhexis during the first and second stage of labor. On multivariable ordinal logistic regression analysis, factors independently associated with the length of the first stage of labor were the type of neuraxial analgesia, gestational age, fetal weight, labor induction, and parity (Supplementary Table 1). Patients who underwent EA were 1.32 times more likely to have a longer first stage of labor (95% CI 1.06–1.64, *p* = 0.012) than those patients who received SEA. Regarding oxytocin acceleration of the first stage of labor, factors independently associated with labor augmentation were fetal weight, multiparity, Asian ethnicity, and induction of labor. The type of neuraxial analgesia and oxytocin augmentation had a non-statistically significant association: patients who underwent EA were 1.28 times more likely to have the first stage of labor augmented with oxytocin (95% CI 0.99–1.63; *p* = 0.058) than those patients who received SEA (Supplementary Table 2). After stratifying the study population per oxytocin augmentation in the first stage of labor, the type of neuraxial analgesia was associated with the first stage of labor length only in those patients who did not receive oxytocin (Supplementary Table 3 and 4; multivariable ordinal logistic regressions; oxytocin augmentation during the first stage OR 0.97 (95% CI 0.68–1.41, *p* = 0.883); labor not augmented with oxytocin during the first stage OR 1.59 (95% CI 1.21–2.08, *p* = 0.001)).

**Table 2:** Labor characteristics, overall and stratified according to the type of neuraxial analgesia

Variable	Total (1499)	EA (402)	SEA (1097)	*p* value
Cervical dilation at analgesia administration (cm, IQR)	4 (4–5) Missing: 0	4 (4–5)	4 (4–5)	0.327
Cervical dilation = 4 cm at analgesia administration (*n *%)	1499 Missing: 0	210 (52.2)	551 (50.3)	0.462
Cervical dilation = 5 cm at analgesia administration (*n *%)		128 (31.8)	341 (31)	
Cervical dilation = 6 cm at analgesia administration (*n *%)		64 (16)	205 (18.7)	
Need for augmentation with oxytocin during the first stage (*n *%)	519 (34.6) Missing: 0	159 (39.6)	360 (32.8)	0.017
Need for augmentation with oxytocin during the second stage (*n *%)	458 (30.5) Missing: 0	138 (34.3)	320 (29.2)	0.059
Amniorrhexis performed during the first stage (*n *%)	315 (21) Missing: 0	90 (22.4)	225 (20.5)	0.438
Amniorrhexis performed during the second stage (*n *%)	31 (2) Missing: 0	6 (1.5)	25 (2.3)	0.341
Duration of the first stage of labor (min)				
Median (IQR) (total: 1379, missing:2)	255 (165–405)	300 (180–420) Missing: 1	240 (150–390) Missing: 1	0.004
Mean (95% CI) (total:1379, missing: 2)	295 (167)	315 (171) Missing: 1	287 (164) Missing: 1	
Duration of Transition phase (min)				
Median (IQR) (total:1377, missing: 2)	15 (0–45)	15 (0–30) Missing: 1	15 (0–45) Missing: 1	0.366
Mean (95% CI) (total:1377, missing: 2)	29 (38)	26 (32) Missing: 1	31 (40) Missing: 1	
Duration of the active second stage of labor (min)				
Median (IQR) (total: 1312, missing: 2)	50 (29.5–90)	52 (29.5–90) Missing: 1	50 (29.5–89.5) Missing: 1	0.561
Mean (95% CI) (total:1312, missing: 2)	63 (46)	64 (47) Missing: 1	62 (46) Missing: 1	
Total duration of analgesia (min, IQR)	280 (165–430) Missing: 0	287.5 (176–431)	279 (163–425)	0.510
Total duration of labor (min)				
Median (IQR) (total: 1312, missing: 2)	358 (230–510)	385 (240–520) Missing: 1	346.5 (223–503.5) Missing: 1	0.062
Mean (95% CI) (total: 1312, missing: 2)	380 (190)	397 (195) Missing: 1	374 (188) Missing: 1	

*CI* confidence interval, *IQR* interquartile range, *min* minutes, *EA* epidural analgesia, *SEA* spinal epidural analgesia

Characteristics of delivery and maternal outcomes are reported in Table 3. No statistically significant differences were observed in total blood loss, in the proportion of patients who developed post-partum hemorrhage, CS rate, or vacuum delivery.

**Table 3:** Mode of delivery and obstetrics outcomes, overall and compared between the type of neuraxial analgesia

Variable	N TOT (missing)	EA (402)	SEA (1097)	*p* value
Blood loss (mL, IQR)	300 (200–500) Missing: 0	300 (200–500)	300 (200–500)	0.679
PPH ≥ 1000 mL (*n*.%)	102 (6.8) Missing: 0	26 (6.5)	76 (7)	0.756
Severe PPH (> 2000 mL) (*n *%)	3 (0.2) Missing: 0	0 (0)	3 (0.3)	0.569
CS (*n *%)	185 (12.3) Missing: 0	57 (14.2)	128 (12)	0.190
CS performed in the first stage (*n *%)	118 (63.7) Missing: 0	39 (68.4)	79 (61.7)	0.381
CS performed in the second stage (*n *%)	67 (36.2) Missing: 0	18 (31.6)	49 (38.2)	0.381
CS due to abnormal FHR	71 (38.4) Missing: 0	25 (43.9)	46 (35.9)	0.306
CS due to labor dystocia (*n *%)	89 (48.1) Missing: 0	23 (40.3)	66 (51.6)	0.159
Emergent CS (*n *%)	26 (14) Missing: 0	6 (10.5)	20 (15.6)	0.357
Vacuum delivery (*n *%)	102 (6.8) Missing: 0	29 (8.4)	73 (7.5)	0.703
Vacuum delivery due to abnormal FHR (*n *%)	38 (37.2) Missing: 0	8 (27.6)	30 (41.1)	0.203
Vacuum delivery due to arrest of progression (*n *%)	44 (43.2) Missing: 0	16 (55.2)	28 (38.4)	0.122
Vacuum delivery due to other indications (*n *%)	20 (19.6) Missing: 0	5 (17.2)	15 (20.5)	0.704
Intact perineum (*n *%)	149 (11.3) Missing: 0	36 (10.4)	116 (11.7)	0.537
Episiotomy (*n *%)	264 (20.1) Missing: 0	83 (24.1)	181 (18.7)	0.032
Severe vaginal tears (3rd and 4th degree) (*n *%)	24 (1.8) Missing: 0	5 (1.5)	19 (2)	0.646
Tracheloraphy (*n *%)	19 (1.45) Missing: 0	5 (1.45)	14 (1.44)	1.000
Fundal pressure (*n *%)	76 (5.8) Missing: 0	31 (9)	45 (4.6)	0.003
Fundal pressure due to abnormal FHR (*n* %)	20 (26.3) Missing: 0	5 (16.1)	15 (33.3)	0.097
Fundal pressure due to arrest of progression (*n *%)	41 (53.9) Missing: 0	21 (67.8)	20 (44.5)	0.045
Fundal pressure due to other indications (*n *%)	15 (5.8) Missing: 0	5 (16.1)	10 (22.2)	0.512

*IQR* interquartile range, *EA* epidural analgesia, *SEA* spinal epidural analgesia, *PPH* post-partum hemorrhage, *CS* cesarean section, *FHR* fetal heart rate

Overall, 185/1499 (12.3%) patients underwent CS during labor, 57/402 (14.2%) in the EA group and 128/1097 (11.7%) in the SEA group (*p* = 0.190). One-hundred and eighteen (63.4%) women underwent CS during the first stage of labor, 39/57 (68.4%) in the EA group and 79/128 (61.7%) in the SEA group (*p* = 0.381). Sixty-seven (36.2%) CSs were performed during the second stage of labor, 18/57 (31.6%) in the EA group and 49/128 (38%) in the SEA group (*p* = 0.381). Seventy-one (38.3%) CS were performed due to abnormal fetal heart rate (FHR), 25/57 (43.8%) in the EA group and 46/128 (35.9%) in the SEA group, whereas 89 (48.1%) CS were performed due to labor dystocia, 23/57 (40.3%) in the EA group and 66/128 (51–6%) in the SEA group (*p* = 0.159). No statistically significant difference between the two groups was noted in urgency level according to the classification of Lucas: 6/57 (10.5%) and 20/128 (15.6%) emergent CS were performed in the EA and SEA group, respectively (*p* = 0.357) [12].

Vaginal delivery was achieved by 1314/1499 (87.7%) women; 102/1314 (7.8%) required vacuum extraction, 29/345 (8.4%) patients in the EA group, and 73/969 (7.5%) in the SEA group (*p* = 0.603). No differences were noted in severe vaginal tear or trachelorrhaphy in the two groups, whereas more episiotomies and fundal pressure maneuvers were performed in the EA group (Table 3). A multivariable logistic regression confirmed an association between type of neuraxial analgesia and need for fundal pressure, with SEA having a negative association (Supplementary Table 5; OR 0.55, 95% CI 0.34–0.9, *p* = 0.017). Conversely, factors independently associated with episiotomy were higher patient age, nulliparity, fundal pressure, augmentation with oxytocin, fetal birth weight, vacuum delivery, and Asian ethnicity, but not the type of epidural analgesia (Supplementary Table 6; OR 0.52, 95% CI 0.52–1.07, *p* = 0.108).

Regarding newborn outcomes, the median APGAR score at the 1st minute of life was found to be higher in the SEA group, whereas no differences were observed in the APGAR score at the 5th minute of life (Table 4). Median umbilical pH was noted to be higher in the SEA group (7.23, IQR 0.11) than in the EA group (7.22, IQR 0.12; the difference between medians 0.01), whereas median umbilical arterial base excess did not differ between the two groups. A higher proportion of fetuses with acidosis at birth (umbilical artery blood with pH < 7.00 and base deficit > 12 mmol/L according to the International Federation of Gynecology and Obstetrics (FIGO) criteria) was found in the EA group, 27 cases (7.4%) in the EA group and 49 cases (4.9%) in the SEA group, but the difference did not reach statistical significance (*p* = 0.075) [13]. A multivariable logistic regression (Supplementary Table 7) confirmed the lack of association between fetal acidosis and the type of neuraxial analgesia.

**Table 4:** Fetal outcomes, overall, and compared between the type of neuraxial analgesia

Variable	N TOT (1499)	EA (402)	SEA (1097)	*P*-value
Fetal weight at birth (g, SD)	3362 (430) Missing: 33	3339 (434) Missing 7	3371 (428) Missing 26	0.201
Fetal weight at birth > 4000 g (*n *%)	103 (7) Missing: 33	26 (6.6) Missing 7	77 (7.2) Missing 26	0.686
Fetal weight at birth < 2500 g (*n *%)	34 (2.3) Missing: 33	9 (2.3) Missing 7	25 (2.3) Missing 26	0.950
Umbilical arterial pH (pH, IQR)	7.23 (7.17–7.29) Missing: 127	7.22 (7.16–7.28) Missing 35	7.23 (7.18–7.29) Missing 92	0.034
Umbilical arterial base excess (mmol/L, IQR)	– 5.6 (– 8 to – 3.5) Missing: 126	– 5.8 (– 3.6 to – 8.4) Missing 35	– 5.5 (– 3.5 to – 7.9) Missing 92	0.529
Acidosis according to ACOG (*n *%)	76 (5.5) Missing: 126	27 (7.4) Missing 35	49 (4.9) Missing 91	0.075
Umbilical arterial pH < 7 (*n *%)	19 (1.4) Missing: 127	11 (3) Missing 35	8 (0.8) Missing 92	0.002
Umbilical arterial base excess < – 12 (*n *%)	76 (5.5) Missing: 126	27 (7.4) Missing 35	49 (4.9) Missing 91	0.075
APGAR at 1st minute (IQR)	9 (9–10) Missing: 1	9 (9–9) Missing: 1	9 (9–10) Missing: 0	0.036
APGAR at 5th minute (IQR)	10 (10–10) Missing: 4	10 (10–10) Missing 2	10 (10–10) Missing 2	0.113
APGAR at 5th minute < 7 (*n *%)	12 (0.8) Missing: 4	4 (1) Missing 2	8 (0.7) Missing 2	0.743

*EA* epidural analgesia, *SEA* spinal epidural analgesia, *g* grams, *SD* standard deviation, *ACOG* American College of Obstetricians and Gynecologists

## Discussion

In the present study, a shorter first stage of labor was observed among patients who underwent SEA than among those who received EA and higher use of the fundal pressure maneuver during the second stage in the EA group. These findings suggest that the neuraxial analgesia technique may influence labor dynamic and duration as observed by three previous studies [14–16].

To date, six studies have investigated differences in labor length between patients who underwent SEA or EA. Consistently with our findings, Frigo et al., Wang et al., and Tsen et al., two observational studies and one randomized controlled trial, respectively, observed a shorter first stage of labor in patients who underwent SEA as compared to EA [14–16]. Conversely, no differences between the two techniques in labor length were reported by Poma et al., although they observed an earlier start of oxytocin use in the EA than the SEA group [17]. These differences may be explained by the inclusion in Poma’s study of patients who underwent neuraxial analgesia at a cervical dilation < 3 cm, which may have masked the effects of the neuraxial analgesia on the labor length [17]. In a randomized controlled trial on 2183 patients, Norris et al. found no differences in the first stage of labor between EA and SEA; however, the study has been criticized because all patients underwent a lidocaine–epinephrine test dose, which might have counterbalanced any technique-related effect on uterine activity [18]. A comparative study between EA and SEA conducted by Cortes et al. found no statistically significant disparities regarding the length of the first stage. However, the small study sample size limited the study’s power [19].

Our and previous results suggest that neuraxial analgesia exerts some effects on uterine activity and labor duration. These effects have been related to the significant reduction in plasmatic catecholamines caused by the labor analgesia, although a significant reduction in plasmatic levels was observed for epinephrine but not norepinephrine [20]. Since epinephrine and norepinephrine exert competing effects on uterine contractions, a reduction in circulating epinephrine after regional analgesia may result in a substantial increase in uterine activity [21]. As suggested by Cascio et al., the rapid fall in circulating epinephrine after intrathecal opioid administration may cause a sudden change in circulating catecholamines, resulting in augmentation of uterine activity and, possibly, faster cervical dilatation rate [11]. Besides a direct effect on myometrium contractility, catecholamine changes after neuraxial analgesia are also thought to reduce hyperventilation and therefore indirectly improve uterine perfusion and contractile activity [21, 22]. These effects may explain differences in oxytocin use and first stage length since catecholamine changes were more evident after SEA due to faster pain relief [7]. Notably, our data show a statistically significant difference in the first stage length only in the subgroup without oxytocin augmentation. In addition to the first stage length, catecholamine changes may explain the observed lower use of fundal pressure maneuver for the arrest of the descent part progression in the SEA group. However, this finding may be further explained by the higher grade of motor blockade associated with EA, which may result in poorer mobility and decreased pelvic muscle tone in the laboring woman [14, 18, 23], and by the higher degree of sacral spread and improved sacral dermatome coverage observed in the SEA [24]. Other labor and delivery characteristics, including maternal outcomes, did not differ, consistent with findings in the literature [7].

Regarding newborn outcomes, we observed a higher mean fetal arterial pH in the SEA group, although not clinically significant (7.22 vs 7.23). Moreover, despite fewer cases of fetal arterial pH < 7 in the SEA group, the prevalence of acidosis according to the FIGO criteria was not statistically significantly different (7.4% in EA vs 4.9% in SEA, *p* = 0.074) [15]. The multivariable logistic regression confirmed that the neuraxial analgesia technique does not appear to be associated with fetal acidosis, as previously reported [7, 25]. Noteworthy, a recent metanalysis showed a higher risk of non-reassuring fetal heart rate tracing in patients who underwent SEA [26]. This observation was explained by the predominance of alpha-adrenergic activity after the rapid analgesic effect of SEA resulting in uterine hyperstimulation [27]. Nevertheless, fetal outcomes were not investigated in the metanalysis, and the higher rate of non-reassuring fetal heart rate tracings may have no clinical relevance [26]. Notably, we did not observe a higher rate of CS for non-reassuring fetal heart rate tracing in patients who underwent SEA. Moreover, this meta-analysis did not consider that fetal heart rate tracing abnormalities associated with EA usually occur later than those after SEA analgesia, resulting in a possible underestimation. Finally, the large variety in drugs and doses adopted both in EA and in SEA in previous studies might have resulted in a more heterogeneous fetal response to neuraxial analgesia.

## Strength and limitations

Several limitations in the present study should be considered when interpreting the results. The retrospective design precluded accessing data regarding uterine activity and fetal heart tracing, VAS score, patients’ satisfaction, and technique’s complications (nausea, vomiting, maternal hypotension, itching, dural puncture, and motor blockade). Moreover, due to the observational design of the study, with no randomization of patients in the two groups, possible confounding factors cannot be excluded and that may have influenced the association between the neuraxial analgesia technique and investigated outcomes. Notably, the retrospective design does not allow achieving conclusions regarding causality, regardless of biological plausibility. However, some strengths support the reliability of our observations. Although retrospective in design, most analyzed variables were prospectively collected by gynecologists and anesthesiologists in a dedicated database, supporting the completeness and correctness of data collection. Moreover, the inclusion of women who received neuraxial analgesia at the beginning of the active phase of labor allows standardizing the starting point, excluding a possible confounding effect related to a less reliable length of the prodromic phase. Finally, the present study has two strengths with respect to the analgesia techniques. First, SEA and EA were performed per a standardized and shared protocol by a limited group of dedicated anesthesiologists, guaranteeing homogeneity in the technique. Second, which technique was used was based on the anesthesiologist’s preference, a personal attitude. Since women were assigned by chance to the anesthesiologist on duty, EA and SEA group assignments may be considered pseudo-random.

## Conclusions

SEA appears to be associated with a shorter length of the first stage of labor and a lower rate of fundal pressure use than EA does. These observations are consistent with two previous studies and a biochemical plausibility, supporting the SEA as a possible preferred technique for pain relief during labor. However, we did not observe differences in maternal or neonatal outcomes, questioning whether the effects of SEA on labor dynamic may be clinically relevant. Therefore, further studies are needed to clarify differences between SEA and EA analgesia in terms of labor dynamic and in terms of maternal and fetal outcomes before concluding that one technique is superior to the other.

## Supplementary Information

Below is the link to the electronic supplementary material.

Supplementary file1 (DOCX 41 KB)

Supplementary file1 (DOCX 41 KB)
