# Supplementary material for: Effects of neuraxial analgesia technique on labor and maternal–fetal outcomes: a retrospective study
Source: Arch Gynecol Obstet. 2022 May 22;307(4):1233–41. doi: 10.1007/s00404-022-06600-6 (PMC10023596; doi:10.1007/s00404-022-06600-6)
Supplement: Supplementary file 1 — Supplementary file1 (DOCX 41 KB) [file 404_2022_6600_MOESM1_ESM.docx]

**Supplementary Table legend**

**Supplementary** **Table 1.** Multivariable ordinal regression to investigate factors that influence the first stage length.

**Supplementary** **Table 2.** Multivariable ordinal regression to investigate factors that influence oxytocin augmentation during the first stage.

**Supplementary** **Table 3.** Multivariable ordinal regression to investigate factors that influence first stage length among patients who underwent labor augmentation with oxytocin during the first stage.

**Supplementary** **Table 4.** Multivariable ordinal regression to investigate factors that influence first stage length among patients who did not undergo labor augmentation with oxytocin during the first stage.

**Supplementary Table 5.** Multivariable Logistic Regression to investigate risk factors for fundal pressure.

**Supplementary** **Table 6.** Multivariable Logistic Regression to investigate risk factors for episiotomy.

**Supplementary Table 7.** Logistic Regression to investigate risk factors for acidosis at birth.

| **Supplementary** **Table 1. Multivariable ordinal regression to investigate factors that influence the first stage length.** | | | | |
| --- | --- | --- | --- | --- |
| **Duration of the first stage of labor** | **OR** | **[95% Conf. Interval]** | | **p** |
| **Type of neuraxial analgesia (SEA vs EA)** | 0.76 | 0.61 | 0.94 | 0.012 |
| **Age** | 1.01 | 0.99 | 1.03 | 0.314 |
| **Gestational age** | 1.13 | 1.05 | 1.22 | 0.002 |
| **Fetal weight** | 1.00 | 1.00 | 1.00 | 0.000 |
| **BMI > 30 kg/m2** | 1.40 | 0.88 | 2.23 | 0.149 |
| **Multiparous** | 0.34 | 0.27 | 0.44 | 0.000 |
| **Asiatic** | 1.81 | 1.02 | 3.23 | 0.042 |
| **Induction of labor** | 0.53 | 0.42 | 0.67 | 0.000 |
| **Cervical Dilation at analgesia administration** | 0.95 | 0.83 | 1.07 | 0.379 |
| **Oxytocin augmentation during the first stage** | 7.18 | 5.70 | 9.03 | 0.000 |
| **Hypertensive Disorders** | 0.93 | 0.60 | 1.45 | 0.760 |
| **Diabetes** | 1.05 | 0.69 | 1.56 | 0.831 |
| **IUGR** | 0.71 | 0.32 | 1.56 | 0.403 |
| **Oligohydramnios** | 0.93 | 0.58 | 1.48 | 0.760 |
| **Polyhydramnios** | 1.92 | 0.80 | 4.62 | 0.143 |
| **Cholestasis** | 3.58 | 0.96 | 13.37 | 0.058 |
| **Fetal Malformation** | 1.32 | 0.42 | 4.17 | 0.631 |
| OR, odds ratio; SEA, spine epidural analgesia; EA, epidural analgesia; BMI, body mass index; IUGR, intrauterine restriction | | | | |

| **Supplementary** **Table 2. Multivariable ordinal regression to investigate factors that influence oxytocin augmentation during the first stage.** | | | | |
| --- | --- | --- | --- | --- |
| **Oxytocin augmentation during the first stage** | **OR** | **[95% Conf. Interval]** | | **p** |
| **Type of neuraxial analgesia (SEA vs EA)** | 0.78 | 0.61 | 1.01 | 0.058 |
| **Age** | 1.01 | 0.99 | 1.03 | 0.392 |
| **Gestational age** | 1.01 | 0.92 | 1.11 | 0.771 |
| **Fetal weight** | 1.00 | 1.00 | 1.00 | 0.000 |
| **BMI > 30 kg/m2** | 1.60 | 0.95 | 2.70 | 0.079 |
| **Multiparous** | 0.37 | 0.27 | 0.51 | 0.000 |
| **Asiatic** | 3.58 | 1.83 | 7.02 | 0.000 |
| **Induction of labor** | 1.88 | 1.45 | 2.44 | 0.000 |
| **Cervical Dilation at analgesia administration** | 0.86 | 0.74 | 1.67 | 0.061 |
| **Hypertensive Disorders** | 0.95 | 0.57 | 1.57 | 0.839 |
| **Diabetes** | 1.46 | 0.91 | 2.32 | 0.113 |
| **IUGR** | 0.56 | 0.21 | 1.60 | 0.286 |
| **Oligohydramnios** | 1.18 | 0.69 | 2.02 | 0.535 |
| **Polyhydramnios** | 1.30 | 0.52 | 3.27 | 0.570 |
| **Cholestasis** | 0.82 | 0.19 | 3.53 | 0.785 |
| **Fetal Malformation** | 0.85 | 0.21 | 3.47 | 0.822 |
| OR, odds ratio; SEA, spine epidural analgesia; EA, epidural analgesia; BMI, body mass index; IUGR, intrauterine restriction | | | | |

| **Supplementary** **Table 3. Multivariable ordinal regression to investigate factors that influence first stage length among patients who underwent labor augmentation with oxytocin during the first stage.** | | | | |
| --- | --- | --- | --- | --- |
| **First stage length** | **OR** | **[95% Conf. Interval]** | | **P** |
| **Type of neuraxial analgesia (SEA vs EA)** | 1.03 | 0.71 | 1.48 | 0.883 |
| **Age** | 1.01 | 0.97 | 1.04 | 0.582 |
| **Gestational age** | 1.08 | 0.94 | 1.24 | 0.282 |
| **Fetal weight** | 1.00 | 0.99 | 1.00 | 0.093 |
| **BMI > 30 kg/m2** | 1.87 | 0.95 | 3.67 | 0.070 |
| **Multiparous** | 0.34 | 0.20 | 0.59 | 0.000 |
| **Asiatic** | 1.42 | 0.67 | 3.02 | 0.358 |
| **Induction of labor** | 0.52 | 0.36 | 0.77 | 0.001 |
| **Cervical Dilation at analgesia administration** | 0.96 | 0.77 | 1.20 | 0.737 |
| **Hypertensive Disorders** | 1.04 | 0.50 | 2.18 | 0.904 |
| **Diabetes** | 1.25 | 0.66 | 2.37 | 0.498 |
| **IUGR** | 0.74 | 0.14 | 3.86 | 0.724 |
| **Oligohydramnios** | 1.03 | 0.48 | 2.17 | 0.945 |
| **Polyhydramnios** | 1.07 | 0.31 | 3.71 | 0.910 |
| **Cholestasis** | 0.50 | 0.68 | 3.63 | 0.490 |
| **Fetal Malformation** | 0.15 | 0.00 | 10.74 | 0.380 |
| OR, odds ratio; SEA, spine epidural analgesia; EA, epidural analgesia; BMI, body mass index; IUGR, intrauterine restriction | | | | |

| **Supplementary** **Table 4. Multivariable ordinal regression to investigate factors that influence first stage length among patients who did not undergo labor augmentation with oxytocin during the first stage.** | | | | |
| --- | --- | --- | --- | --- |
| **First stage length** | **OR** | **[95% Conf. Interval]** | | **p** |
| **Type of neuraxial analgesia (SEA vs EA)** | 0.63 | 0.48 | 0.82 | 0.001 |
| **Age** | 1.01 | 0.99 | 1.04 | 0.212 |
| **Gestational age** | 1.16 | 1.05 | 1.27 | 0.002 |
| **Fetal weight** | 1.00 | 1.00 | 1.00 | 0.002 |
| **BMI > 30 kg/m2** | 1.17 | 0.62 | 2.21 | 0.630 |
| **Multiparous** | 0.34 | 0.26 | 0.45 | 0.000 |
| **Asiatic** | 2.17 | 0.86 | 5.48 | 0.100 |
| **Induction of labor** | 0.53 | 0.39 | 0.71 | 0.000 |
| **Cervical Dilation at analgesia administration** | 0.93 | 0.80 | 1.08 | 0.371 |
| **Hypertensive Disorders** | 0.87 | 0.50 | 1.53 | 0.634 |
| **Diabetes** | 0.93 | 0.54 | 1.60 | 0.805 |
| **IUGR** | 0.68 | 0.27 | 1.71 | 0.412 |
| **Oligohydramnios** | 0.82 | 0.45 | 1.50 | 0.531 |
| **Polyhydramnios** | 3.41 | 0.99 | 11.81 | 0.053 |
| **Cholestasis** | 10.17 | 2.20 | 46.96 | 0.003 |
| **Fetal Malformation** | 1.90 | 0.53 | 6.69 | 0.329 |
| OR, odds ratio; SEA, spine epidural analgesia; EA, epidural analgesia; BMI, body mass index; IUGR, intrauterine restriction | | | | |

| **Supplementary Table 5. Multivariable Logistic Regression to investigate risk factors for fundal pressure.** | | | | |
| --- | --- | --- | --- | --- |
| **Fundal Pressure** | **OR** | **[95% Conf. Interval]** | | **p** |
| **Type of neuraxial analgesia (SEA vs EA)** | 0.55 | 0.34 | 0.9 | 0.017 |
| **Age** | 1 | 0.96 | 1.06 | 0.619 |
| **Gestational Age** | 1.14 | 0.92 | 1.41 | 0.219 |
| **Multiparity** | 0.12 | 0.03 | 0.51 | 0.004 |
| **BMI>30 kg/m^2^** | 0.78 | 0.23 | 2.68 | 0.699 |
| **Obstetric complications** | 1.82 | 0.96 | 3.48 | 0.067 |
| **Induced Labor** | 0.74 | 0.42 | 1.30 | 0.294 |
| **Augmentation with oxytocin** | 4.13 | 2.26 | 7.53 | 0.000 |
| **Fetal weight** | 1 | 0.99 | 1.00 | 0.974 |
| OR, odds ratio; SEA, spine epidural analgesia; EA, epidural analgesia; BMI, body mass index. | | | | |

| **Supplementary Table 6. Multivariable Logistic Regression to investigate risk factors for episiotomy.** | | | | |
| --- | --- | --- | --- | --- |
| **Episiotomy** | **OR** | **[95% Conf. Interval]** | | **p** |
| **Type of neuraxial analgesia (SEA vs EA)** | 0.75 | 0.52 | 1.07 | 0.108 |
| **Age** | 1.06 | 1.02 | 1.09 | 0.002 |
| **Gestational Age** | 0.97 | 0.84 | 1.10 | 0.606 |
| **Multiparity** | 0.30 | 0.18 | 0.51 | 0.000 |
| **BMI>30 kg/m^2^** | 0.67 | 0.29 | 1.55 | 0.347 |
| **Fundal Pressure** | 4.92 | 1.69 | 4.63 | 0.000 |
| **Obstetric Complications** | 1.08 | 0.26 | 0.32 | 0.751 |
| **Induced Labor** | 0.97 | 1.19 | 0.17 | 0.864 |
| **Augmentation with oxytocin** | 2.27 | 0.4 | 4.63 | 0.000 |
| **Fetal weight** | 1.00 | 0.0002 | 2.05 | 0.041 |
| **Vacuum delivery** | 15.38 | 4.75 | 8.85 | 0.000 |
| **Asiatic** | 4.32 | 1.74 | 3.62 | 0.000 |
| OR, odds ratio; SEA, spine epidural analgesia; EA, epidural analgesia; BMI, body mass index. | | | | |

| **Supplementary Table 7. Logistic Regression to investigate risk factors for acidosis at birth.** | | | | |
| --- | --- | --- | --- | --- |
| **ACIDOSIS (FIGO)** | **OR** | **[95% Conf. Interval]** | | **p** |
| **Type of neuraxial analgesia (SEA vs EA)** | 0.72 | 0.42 | 1.21 | 0.214 |
| **Age** | 1.02 | 0.97 | 1.07 | 0.495 |
| **Gestational age** | 1.12 | 0.91 | 1.39 | 0.272 |
| **Multiparity** | 1.01 | 0.48 | 2.11 | 0.987 |
| **BMI>30 kg/m2** | 1.49 | 0.54 | 4.12 | 0.440 |
| **Obstetric complications** | 0.79 | 0.38 | 1.64 | 0.523 |
| **Induction of Labor** | 1.50 | 0.84 | 2.68 | 0.171 |
| **Need for augmentation with oxytocin** | 0.78 | 0.43 | 1.40 | 0.400 |
| **Duration of the first stage of Labor (min)** | 1.00 | 0.99 | 1.00 | 0.199 |
| **Duration of Transition phase (min)** | 0.99 | 0.98 | 1.00 | 0.059 |
| **Duration of the second stage of Labor (min)** | 1.01 | 1.00 | 1.01 | 0.000 |
| **Fetal Weight** | 0.99 | 0.99 | 1.00 | 0.199 |
| OR, odds ratio; SEA, spine epidural analgesia; EA, epidural analgesia; BMI, body mass index | | | | |
